# SGPL1_321_ mutation: one main trigger for invasiveness of pediatric alveolar rhabdomyosarcoma

**DOI:** 10.1038/s41417-019-0132-8

**Published:** 2019-08-27

**Authors:** Anna Adamus, Nadja Engel, Guido Seitz

**Affiliations:** 1 Department of Pediatric Surgery, University Hospital Marburg, Baldingerstrasse, 35033 Marburg, Germany; 2 Department of Oral and Maxillofacial Surgery, Facial Plastic Surgery, Rostock University, Medical Center, Schillingallee 35, 18057 Rostock, Germany

**Keywords:** Biomarkers, Biomarkers, Cancer metabolism, Paediatric cancer

## Abstract

Sphingosine-1-phosphate (S1P), a sphingolipid with second messenger properties, is a main regulator of various cellular processes including lymphocyte cell trafficking, angiogenesis, cell proliferation, and survival. High S1P concentrations and deficiencies in S1P degradation have been associated with cancer cell progression, their directed chemoattraction and promotion of chemo-resistance mechanism. The endoplasmic reticulum (ER) membrane localized enzyme sphingosine-1-phosphate lyase (SGPL1) has a key role in prevention of S1P overstimulation in tumor cells by its irreversible S1P degradation activity. In this paper we demonstrated a SGPL1 overexpression and mislocalization in pediatric alveolar rhabdomyosarcoma (RMA) cells. Moreover, a homozygous point mutation from A to G at position 321 in the coding sequence was obvious, which interferes with the S1P degradation activity and correct localization in the ER-membrane. By complementation with the native SGPL1 variant, the ER localization was restored in RMA cells. More importantly, the SGPL1 restauration prevents the S1P induced migration and colony formation of RMA cells, significantly. This observation opens new highways for the treatment of pediatric RMA by gene therapeutic SGPL1 renewal and recommends the detection of specific SGPL1 mutations as pathological, molecular metastasis marker.

## Introduction

Besides glycerophospholipids and cholesterol, sphingolipids are the main components of the cell membrane [1]. All sphingolipids are basically built from the unsaturated amino alcohol: sphingosine. The simplest sphingolipid is ceramide which can be converted into sphingosine by a ceramidase and reconverted by a ceramide synthetase activity [2, 3]. By the enzyme activity of the sphingosine kinase 1 and 2 (SPHK1, 2), sphingosine can be further phosphorylated to the signaling lipid sphingosine-1-phosphate (S1P) [2, 3]. The reaction is reversible by the dephosphorylation activity of two specific S1P phosphatases (SPP1 or SPP2) [2, 3].

The second messenger S1P is a potent and ubiquitous sphingosine-based signaling molecule which is mainly produced, transported and transferred in extracellular fluids such as blood and intestinal liquids. Autocrine or paracrine S1P signaling is mediated by the action of five specific G-protein-coupled receptors (named S1PR_1,2,3,4,5_) which are regulating various processes and are expressed in a tissue and cell specific pattern [4–6]. The S1P concentration is ranging from 0.2 to 1.1 µM in the human blood and is in contrast very low in human tissue (<1 ng/mg) [7–10]. There is a dynamical balance between these three sphingolipids which is termed as sphingosine/ceramide-S1P rheostat and determines the fate of cells particularly of tumor cells [11, 12]. Thus, high S1P concentrations have a negative effect on the success of anti-cancer therapies and can promote chemo-resistance by suppressing apoptosis and stimulating survival signaling mechanisms in response to stress signals [11–13]. S1P stimulates a brought panel of cellular processes, e.g., cell proliferation, survival, migration, invasion, angiogenesis, T- and B-cell trafficking (inflammation/ immune response), extracellular matrix protein production as well as cytoskeletal reorganization [2, 12, 14–16]. In contrast, high sphingosine and ceramide levels promote opposite cellular processes such as apoptosis induction, autophagy, ER-stress response, cell cycle arrest and cellular senescence [2, 3, 12]. S1P acts as chemoattractant for immune cells and metastatic tumor cells [14, 17]. Thus, high S1P levels can promote cancer progression and some autoimmune diseases such as multiple sclerosis and allergies [4, 18, 19]. Several cancer entities such as oral squamous cell carcinoma (OSCC), non-small cell lung cancer (NSCLC), hepatocellular carcinoma (HCC), esophageal cancer, pancreatic cancer, erythroleukemia, breast cancer, bladder cancer and colorectal cancer displayed an overexpression of the S1P-synthesizing enzyme SPHK1 which is therefore announced as a potential prognostic tumor marker [15, 20–27]. The prevention of the S1P overstimulation is a major treatment challenge. There are four treatment approaches: (1) disruption of the S1P production by the reduction of the SPHK1/2 activity with competitive inhibitors (e.g., DHS), (2) specific blocking of S1P receptors with S1P agonists, e.g., FTY720 (fingolimod), (3) S1P blocking by a S1P-specific monoclonal antibody (sphingomab) as well as S1P-specific binder (NOX-S93) or (4) reduction of the S1P pool by the enhancement of the sphingosine-1-phosphate lyase activity (SGPL1/ SPL/ SP-lyase; EC 4.1.2.27) [4, 28–31].

The PLP (pyridoxal 5′-phosphate)-dependent enzyme SGPL1 is anchored in the membrane of the endoplasmic reticulum (ER) and plasma membrane as well partly located in the nucleus and cytoplasm [32–34] (https://compartments.jensenlab.org/). It mediates the irreversible cleavage of S1P into the non-sphingolipid phospho-ethanolamine and hexadecenal and thus prevents uncontrolled growth and directed migration of cancer cells [18, 28, 33]. SGPL1 activity is known to support the overcome of chemotherapeutic drug resistance, increase the sensitivity of cells to stress and is ascribed to display a tumor suppressing and anti-oncogenic behavior [3, 35–39]. Own previous studies proved that SGPL1 upregulation is leading to a successful treatment of breast cancer and osteosarcoma [40, 41]. Moreover, we identified a novel SGPL1 localisation in the cytoplasm membrane of primary, epithelial breast cells in vitro, which was missing in several breast cancer cell lines [32]. Furthermore, the S1P stimulated migration of these cancer cells could be diminished by restoring the SGPL1 activity and localization. So far, only a few studies are available for SGPL1 expression and sphingolipid-metabolism in correlation with tumor progression and treatment success in pediatric rhabdomyosarcoma (RMS), which is the most frequent soft tissue sarcoma. Initially, a general SGPL1 expression analysis on transcript and protein level was performed in four RMS cell lines with different histological subtypes (RD, RH-30, HA-OH1, and Ax-OH-1) and one rhabdoid cell line A-204 compared to undifferentiated and differentiated primary myoblasts (HSkM). Further, the SGPL1 distribution and location as well as the function was particularly examined using the two alveolar RMS (RMA) cell lines RH-30 and HA-OH1, which both represent the most aggressive histological RMS subtype. Translocation-positive RMA, especially advanced RMA is accompanied by a significantly worse outcome (event-free 5-year survival is still about 20–30%), mainly by its high metastatic nature and drug resistance mechanism [42]. RMA is characterized by the expression of pax-foxo1 fusion transcription factors which promotes oncogenesis in these cancer type [43]. Our in vitro study presents novel evidence that SGPL1 mediated S1P degradation is an essential target for the prevention of metastasis formation in pediatric alveolar rhabdomyosarcoma.

## Results

### The SGPL1 is overexpressed in alveolar RMS cell lines

**Fig. 1:**
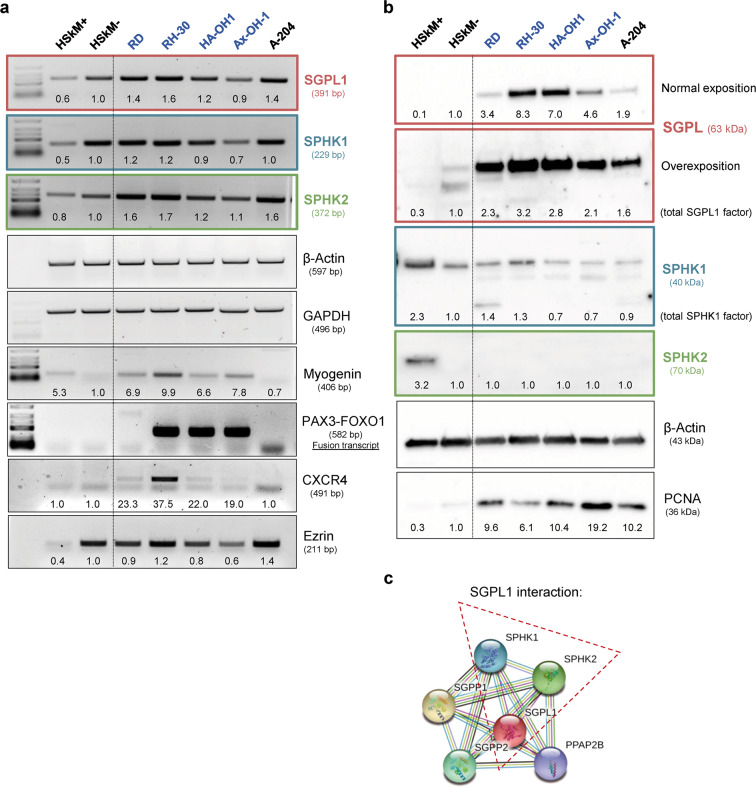
In vitro SGPL1 expression analysis. Evaluation of SGPL1 and SPHK1/2 isoenzyme transcript and protein expression in four RMS cell lines (RME: RD; RMA: RH-30, HA-OH1 and Ax-OH-1) and one rhabdoid cell line (A-204) compared to the non-tumorigenic primary human skeletal myoblasts (HSkM+ = differentiated; HSkM− = undifferentiated). Expression factors were calculated by densitometry and normalized to the HSkM control, which was set to 1. **a** RT-PCR of SGPL1, SPHK1, SPHK2 as well as controls: β-actin and GAPDH (loading control); myogenin and PAX3-FOXO1 (RMS/ myogenic marker); as well as ezrin and CXCR4 (metastasis marker). (Representative images of three independent experiments, *n* = 3). **b** Immuno blots of SGPL1, SPHK1, and SPHK2 as well as β-actin (loading control) and PCNA (proliferation control) (Representative example of three independent experiments, *n* = 3). **c** Network of SGPL1 protein interactions created with, https://string-db.org shows the evidence for co-expression of SGPL1 and SPHK2 (black line) and of SGPL1 interaction with SPHK1/2 (framed in red). All data and download files in STRING are freely available under a ‘Creative Commons BY 4.0’ license

**Fig. 2:**
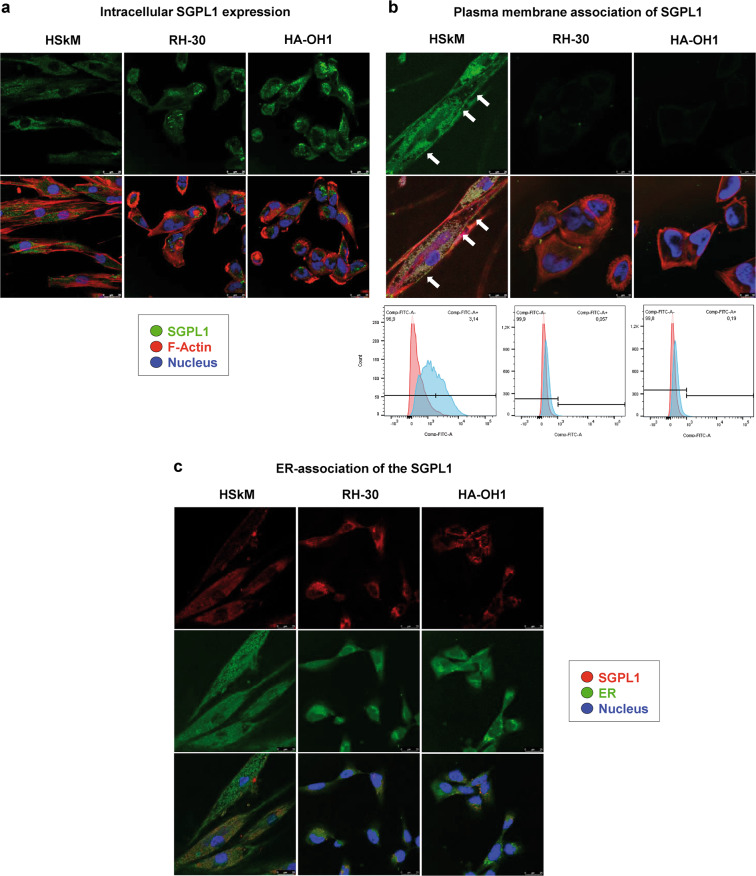
Analysis of cellular SGPL1 expression and distribution. **a** SGPL1 immunofluorescence staining (green) of fixed and permeabilized HSkM, RH-30 and HA-OH cells. Co-labeling of β-actin (red) and nuclei (blue). The RMA cell line RH-30 and HA-OH1 displayed a SGPL1 overexpression and an unusual diffuse cytosolic SGPL1 distribution compared to healthy HSkM cells. HSkM cells showed a SGPL1 distribution around the nucleus. **b** SGPL1 immunofluorescence staining (green) of living (without fixation) HSkM, RH-30 and HA-OH1 cells to label SGPL1 protein in the cytoplasm membrane. Co-labelling of β-actin (red) and nuclei (blue). RH-30 and HA-OH1 RMA cells revealed no co-localization with the cortical actin cytoskeleton and thus no SGPL1 association with the plasma membrane. White arrows mark the SGPL1 protein content which is co-localized with the cortical F-actin fibers in HSkM cells. Determination of SGPL1 association with the plasma membrane by flow cytometry of the RMS cell lines RH-30 and HA-OH1 and compared to HSkM cells. The RMS cells displayed no and HSkM cells a slight plasma membrane association of the SGPL1. **c** SGPL1 immunofluorescence staining (red) and co-labelling with nuclei (blue) and cell permanent ER-tracker endoplasmic reticulum of healthy HSkM cells in comparison with RH-30 and HA-OH1 RMA cell lines. HSkM cells revealed a SGPL1 association with the ER, whereas both RMA cell lines displayed no co-localization with the ER

The general SGPL1 expression signature of pediatric rhabdomyosarcoma cells was analyzed using four established RMS cell lines: RD (representing the embryonal RMS subtype), RH-30, HA-OH1 and Ax-OH-1 (representing the alveolar RMS subtype with PAX3-FOXO1 translocation) (Fig. 1). Their SGPL1 expression features were compared with non-tumorigenic primary human skeletal myoblasts (HSkM-: undifferentiated and HSkM+: differentiated) and the rhabdoid cell line A-204 which is an invasive RMS-like cell line. The undifferentiated human skeletal myoblast cell line (HSkM-) served as control cell line. The HSkM-cells are proliferating and more metabolically active than differentiated HSkM+ cells. On transcript level (Fig. 1a), the SGPL1 expression was slightly increased in the RMS cell lines. Differentiated HSkM+ cells showed the lowest SGPL1 transcript level. Furthermore, expression analysis of both S1P-synthesizing SPHK1/2 isoenzymes revealed almost no altered SPHK1 expression level in the RMS cell lines. The SGPL1-coexpressing enzyme SPHK2 revealed the same expression pattern as it was determined for SGPL1. GAPDH and β-actin amplification were used as loading and housekeeping controls, whereas PAX3-FOXO1 (RMA marker), myogenin (myogenic marker), CXCR4 and ezrin (both metastasis marker for RMS) amplification were used as internal control for RMS cells. Protein expression analysis by western blotting (Fig. 1b) verified a significantly boosted SGPL1 protein content in all tumor cell lines especially in the RMA cell lines RH-30 and HA-OH1 compared to the non-tumorigenic HSkM control. Interestingly, the differentiated HSkM+ cells displayed a significantly increased SPHK1 and SPHK2 protein content compared to the undifferentiated HSkM− control and the tumor cell lines. As internal loading controls, ß-actin labeling (Fig. 1b) and stainfree imaging technique (S. Fig. 1a) were used. The expression factors were also determined densitometrically and normalized to the HSkM control. Figure 1c shows the interaction of SGPL1 with the two sphingosine phosphorylating kinases (SPHK1/2), and the black line indicates the co-expression of SPHK2 and SGPL1. Moreover, the SGPL1 overexpression in RMA cell lines was also confirmed by confocal microscopy imaging (Fig. 2a). SGPL1 was located in the cytoplasm of in the RMA cell lines RH-30 and HA-OH1 (Fig. 2a). The annotated association of the SGPL1 with the ER was not confirmed in RH-30 and HA-OH1 (Fig. 2c). In contrast, the SGPL1 association with the ER was verified in the HSkM cells (Fig. 2a and c). Live cell staining as well as flow cytometric analysis revealed a SGPL1 association with the plasma membrane for HSkM cells, only (Fig. 2b and S. Fig. 1d).

### S1P promotes viability and migration in RMA cell lines

**Fig. 3:**
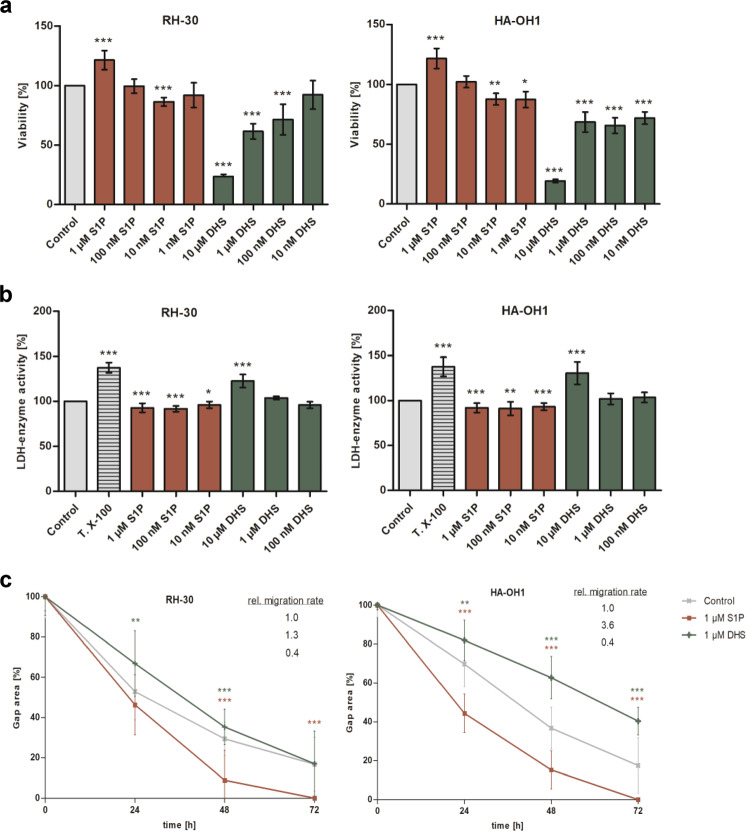
Stimulation of RMA cells with S1P and DHS. **a** Metabolic cell viability was significantly increased in S1P stimulated (green bar) and significantly reduced in DHS stimulated (red bar) RH-30 and HA-OH1 cells compared to the vehicle control (0.1% MeOH). **b** The cell membrane impairment was measured by the release of the cytosolic lactate dehydrogenase (LDH). The vehicle control (0.1% MeOH) was set to 100%. Triton X-100 (T.X-100) treatment functioned as positive control. High DHS concentrations (10 µM) significantly increased (red bar) and the S1P stimulations slightly decreased (green bar) the extracellular LDH activity in supernatants of RH-30 and HA-OH1 cells. **c** Continuous treatment with 1 μM sphingosine-1-phosphate (S1P) significantly enhanced (red line) and 10 µM DHS treatment (green line) significantly reduced the migration speed compared to the vehicle control (0.1% MeOH) in RH-30 and HA-OH1 cells. Mean ± SD, *n* = 8 (MTS- and LDH-assay) and *n* = 4 (wound healing assay), *P < 0.05, **P < 0.01, ***P < 0.001, unpaired *t*-test

RH-30 and HA-OH1 RMA cells were stimulated with concentration series of S1P and the competitive SPHK1/2 inhibitor DL-threo-dihydrosphingosine (DHS) to verify its influence on cellular metabolism, viability and motility (Fig. 3). Continuous stimulation with 1 µM S1P significantly enhanced cell viability of RH-30 and HA-OH1 cells about 20% (Fig. 3a). In contrast, treatment with high DHS concentrations (1 and 10 µM) significantly reduced the cell viability of RMA cell lines about 40 and 80%. Moreover, the lactate dehydrogenase release was increased, which is an indicator for apoptosis induction (Fig. 3b). The migratory activity of RH-30 and HA-OH1 cells was significantly increased under continuous stimulation with 1 µM S1P about 30 and 360%, respectively (Fig. 3c). In contrast, continuous treatment with 1 µM DHS significantly decreased the migratory speed about 60%. In summary, treatment with high concentration of S1P (1 µM) significantly increased the general metabolic activity and migration activity in both RMA cell lines.

### SGPL1 restoration silences the motility and proliferation in RMA cells

**Fig. 4:**
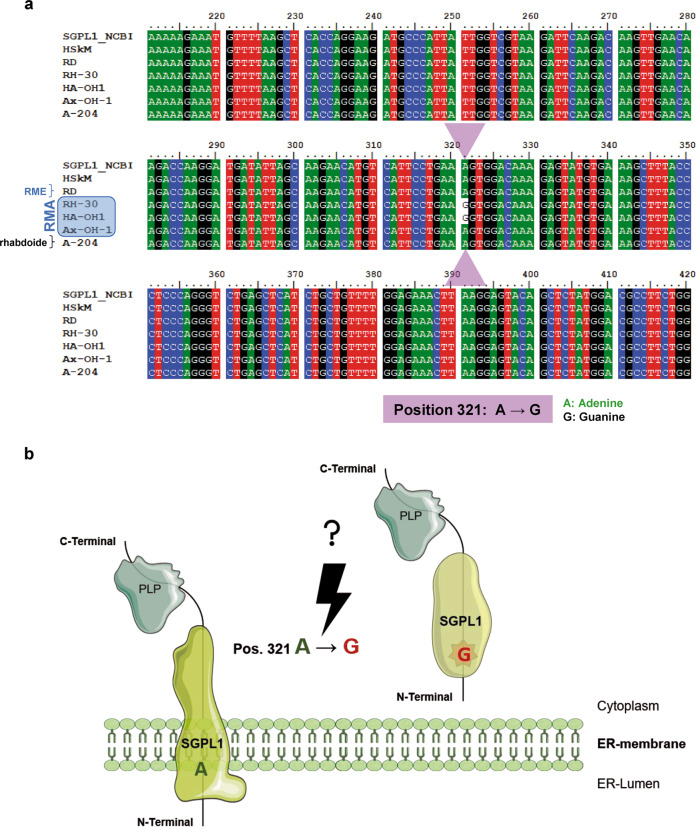
SGPL1 sequence analysis. **a** Nucleotide sequence alignment of the main coding SGPL1 transcript variant 1 between NCBI CDS (Reference Sequence: NM_003901.3) and non-tumorigenic HSkM cells compared to tumor cell lines RD, RH-30, HA-OH1, Ax-OH-1, and A-204. SGPL1 cDNA sequencing revealed a homozygous base exchange from Adenine to Guanine at position 321 in the sequence of the SGPL1 transcript in RH-30 and HA-OH1 RMA cells. **b** Schematically illustration generated with elements of https://smart.servier.com shows the putative effect of the homozygous point mutation in the N-terminal region of the SGPL1. The mutation might interfere with anchorage in the ER membrane resulting in cytoplasmic SGPL1 localization. All data and download files of Servier Medical Art by Servier are freely available at https://smart.servier.com and licensed under a ‘Creative Commons Attribution 3.0 Unported License’

To test the integrity of the SGPL1 sequence of the RMA cell lines, the coding sequence was sequenced first. After forward and reverse sequencing, a site-specific mutation at nucleotide position 321 from A to R was observed. This mutation occurs in the N-terminal domain, representing the transmembrane region. Whether a heterozygous or homozygous mutation is present, the mutated sequence was cloned in the pGEMT vector and ten clones were sequenced again. A homozygous mutation from A to G at position 321 was obvious (Fig. 4a). Figure 4b schematically illustrates the putative effect of the homozygous point mutation in the N-terminal region of the SGPL1 transcript on the protein translation and modification/folding process as well as the correct localization of the SGPL1 protein in the ER-membrane. The mutation might interfere with anchorage in the ER membrane resulting in cytoplasmic SGPL1 localization.

**Fig. 5:**
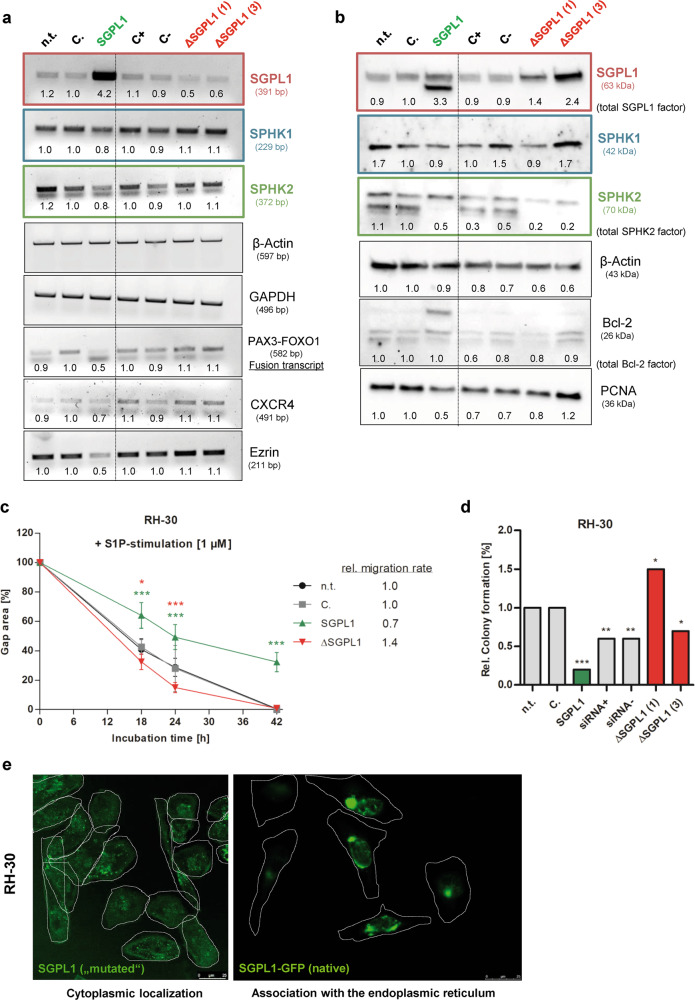
Stable overexpression of native SGPL1. Evaluation of native SGPL1 restauration effects on SGPL1 and SPHK1/2 isoenzyme transcript and protein expression level as well as on cell physiology and SGPL1 localization, representatively in transfected RH-30 cells. The expression factors were all determined densitometrically and normalized to the mock transfection control (C.), which was set to 1. (legend: n.t. = non-transfection control, C. = mock transfection control, SGPL1 = SGPL1 restauration, siRNA+ = positive siRNA control, siRNA− = negative siRNA control and ΔSGPL1 = SGPL1 siRNA). **a** Transcript expression analysis by RT-PCR of SGPL1, SPHK1, SPHK2 as well as controls: β-actin and GAPDH (loading control); PAX3-FOXO1 (RMA marker); as well as ezrin and CXCR4 (metastasis marker). (Representative images of three independent experiments, *n* = 3). **b** Western blot images of SGPL1, SPHK1, SPHK2 and β-actin (loading control) as well as proliferation marker PCNA and anti-apoptotic protein marker Bcl-2 (Representative images of three independent experiments, *n* = 3). **c** Continuous treatment with 1 µM S1P enhanced migration speed of SGPL1-deficient RH-30 cells (black, gray and red line), whereas SGPL1 overexpression significantly inhibited the migration capacity during continuous S1P stimulation (green line). Mean ± SD, *n* = 4, *P < 0.05, **P < 0.01, ***P < 0.001, significantly different compared to mock transfected cells, unpaired *t*-test. **d** Native SGPL1 overexpression inhibited the ability for adherent colony formation in the presence of S1P, significantly (green bar). Mean ± SD, *n* = 3, *P < 0.05, **P < 0.01, ***P < 0.001, significantly different compared to mock transfected cells, unpaired *t*-test. **e** Immunofluorescence-based analysis of the different localisation and distribution pattern of the mutated SGPL1 (left image: red fluorescence; right image: green fluorescence) and native SGPL1 (restored SGPL1: green GFP signal) in RH-30 cells. Note, the SGPL1 variants are not co-localized. Native SGPL1 overexpression restored the ER-association of SGPL1, whereas the mutated SGPL1 showed a diffuse cytoplasmatic localization

By restoration experiments using the native SGPL1 sequence, the SGPL1_321_ related viability and migration activation by 1 µM S1P should be reverted. Therefore, both RH-30 and HA-OH1 cells were stable transfected with the native SGPL1 cDNA in frame with GFP and a constitutive CMV promoter (S. Fig. 4a). In parallel to the SGPL1 overexpression the SGPL1 content was reduced by siRNA transfection in RH-30 cells (ΔSGPL1). SGPL1 overexpression was confirmed on transcript (Fig. 5a) and on protein level (Fig. 5b). Note that two SGPL1 protein bands occur on western blotting. In contrast SGPL1 siRNA reduced SGPL1 transcript levels but increased SGPL1 protein levels. Moreover, SGPL1 overexpression reduced SPHK2 levels, the RMA driver oncogene PAX3-FOXO1 and the metastatic invasion markers ezrin and CXCR4. Also, the expression of the PCNA (proliferating nuclear antigen) was decreased after insertion of the functional SGPL1 variant whereas the indirect-apoptosis marker Bcl-2 was highly expressed in a different band pattern. As internal loading controls ß-actin labeling (Fig. 5b) and stainfree imaging technique (S. Fig. 1b) were used.

Further we examined if S1P induces the stimulation of migration (Fig. 5c) and independent colony formation in RH-30 cells (Fig. 5d and S. Fig. 5) and if the SGPL1 localization (Fig. 5e) could be reverted. The S1P-mediated migration stimulation was reduced about 30% whereas SGPL1-deficient cells showed an enhancement of the migration speed of 40% (Fig. 5c). Similar results were obtained for the independent colony formation; SGPL1 overexpression decreased the colony formation about 70% whereas SGPL1 siRNA insertion increased the value up to 50%, significantly (Fig. 5d). By confocal microscopy the native SGPL1 and the mutated SGPL1_321_ proteins were visualized and the different cellular distribution of the two SGPL1 variants was verified. The native SGPL1 is associated with the ER and in contrast the mutated SGPL1_321_ is found in the cytoplasm (Fig. 5e). No co-localization was obvious (Fig. 5e and S. Fig. 1e). However, stabile overexpression of the native SGPL1 variant caused the reassociation with the ER (Fig. 5e).

## Discussion

Continuous stimulation with a physiological S1P concentration of 1 µM induced a pro-metastatic phenotype in RMA cells, first described by Schneider et al. [29]. This observation was confirmed by our own studies. Briefly, S1P treatment significantly enhanced the metabolic and migratory activity of the RH-30 and HA-OH1 cells (Fig. 3a and c). In contrast, the stimulation with 1–10 µM DHS, a well characterized competitive SPHK1/2 inhibitor [4], decreased the cell viability and the migratory activity. Furthermore, the stimulation with 10 µM DHS increased the lactate dehydrogenase release due to the membrane impairment because of cell death initiation (Fig. 3b). Low SGPL1 content is described for several cancer entities such as breast cancer, colon cancer, OSCC and melanoma, which are associated with a poor prognosis [26, 32, 38, 39]. Therefore, low SGPL1 levels can be expected in RMA cells because it is well known that the loss of SGPL1 enhances the emergence for malignancy and tumor cell resistance to chemotherapeutic drugs including cisplatin and daunorubicin, which were used in RMS treatment [38, 42]. But our first transcript (Fig. 1a) and protein analysis (Fig. 1b and Fig. 2a) displayed a SGPL1 overexpression in alveolar RMS cell lines compared to the healthy primary myoblast control (HSkM). That is a contradiction to our previous data in breast and bone cancer [32, 40] and to other cancer studies focusing on the SGPL1 in cancer progression [28, 38, 44]. Moreover, a low SGPL1 expression status correlates with a significantly reduced overall survival for many tumor entities but surprisingly not for rhabdomyosarcoma (R2 correlation analysis with the online analysis tool: https://hgserver1.amc.nl/cgi-bin/r2/main.cgi; S. Fig. 6a). Furthermore, in-silico analysis using the online tool R2 for correlation analysis (https://hgserver1.amc.nl/cgi-bin/r2/main.cgi) determined a positive correlation of the PAX-FOXO1 fusion status with the SGPL1 expression (S. Fig. 6b). This observation means that RMAs, which harbor these transcript fusions, often show an increased SGPL1 expression. Another observation was the SGPL1 localization was detected in the cytoplasm of RMA cells. No association could be detected with the ER or the plasma membrane (Fig. 2). One explanation for the loss of cell membrane and ER association as well as the fact, that the high SGPL1 content did not reduce the S1P stimulation is an inhibition of the enzyme activity, often caused by mutations.

By sequencing a base exchange at position 321 in the coding sequence of the SGPL1 in RH-30 and HA-OH1 was detected (Fig. 4). This homozygous point mutation was confirmed by forward and reversed sequencing as well as cloning experiments. This site specific SGPL1_321_ mutation was suspected to be the cause of the miss localization in the cytoplasm and S1P-mediated migration enhancement. Possibly, complete loss-of-function of the SGPL1 activity was caused by this mutation, which prevents the irreversible breakdown of S1P to phospho-ethanolamine and hexadecenal. The SGPL1_321_ mutation was recently described by Carney et al. [45]. They reported that SGPL1_321_ is responsible for the development of a steroid-resistant nephrotic syndrome (SNRS syndrome). Furthermore, the complete SGPL1 loss-of-function was proved in SGPL1_321_ mutants by activity measurements in mouse and human fibroblast cell lysates [46, 47].

The SGPL1_321_ mediated effects were restored by the complementation with native SGPL1 variant by transient and stable overexpression. Thereby the ER association (Fig. 5e) and the effective S1P degradation were restored (Fig. 5c, d). The regained SGPL1 functionality allows the degradation of extracellular S1P. This significantly reduces both the S1P-mediated migration activity and the reformation of independent cell colonies of RMA cells (Fig. 5c, d). In human blood, concentrations up to 1 µM S1P are circulating and thus represent a chemoattractant for tumor cells prone to metastasis. It can be concluded that S1P is a potential chemoattractant to force metastatic invasion of RMS cells.

The altered SGPL1 expression has also an impact on the expression of the PAX3-FOXO1 fusion transcript levels (Fig. 5a and S. Fig. 3b), which underlines the assumption of correlated SGPL1 and PAX-FOXO1 expression (S. Fig. 6b). Furthermore, SGPL1 overexpression showed two SGPL1 protein bands (Fig. 5b), which were remarkably present in the undifferentiated HSkM- cells, too (Fig. 1b). The SGPL1 overexpression lowered the expression status of SPHK2 and the markers for metastatic invasion: CXCR4 and ezrin in RH-30 cells (Fig. 5a). SPHK2 is known to suppress growth and enhances apoptosis. But in contrast, in breast cancer SPHK2 is required for EGF-directed cell movement [48]. Finally, SGPL1 overexpression limits the proliferative capacity of RMA cells demonstrated by decreased expression of the proliferation marker PCNA (Fig. 5b and S. Fig. 2b).

A link between the RMS cell morphology, SGPL1_321_ mutation and invasion potential is conceivable. Restoration of native SGPL1 enzyme activity in RH-30 and HA-OH1 was able to silence the overall motility and proliferative capacity in vitro. The mechanism of action should be further verified in an RMA mouse model. Further, the data on SGPL1_321_ mutation need conformation through investigations with primary RMS tumor materials. In future studies, the role of SGPL1 mutations for the patient outcome and the effectiveness of an anti-cancer treatment should be further evaluated.

### Conclusions

We conclude that the SGPL1 expression as well as mutation status may predict the probability for metastasis formation of pediatric rhabdomyosarcoma and other cancer entities. A high SGPL1 expression status without mutation is associated with low probability of metastasis formation. In contrast, no SGPL1 expression and SGPL1 mutations could be a predictor for a high metastasis probability. Finally, functional SGPL1 activity is essential for effective S1P degradation and is one limiting factor for metastasis control. Therefore, we recommend the examination of the SGPL1 expression and mutation status in RMA as basis for a potential personalized treatment approach.

## Materials and methods

### Cell culture procedure

The RD (RME; CCL-136^TM^) and A-204 (rhabdoid cell line; HTB-82^TM^) cells were obtained from ‘ATCC®’ (www.lgcstandards-atcc.org) and RH-30 (RMA; ACC-489) from the German biological resource bank ‘DSMZ’ (https://www.dsmz.de). The RMA cell lines HA-OH1 and Ax-OH-1 were kindly supported by Prof. Dr. Ewa Koscielniak (Cooperative soft tissue sarcoma-/‘CWS’- study group, Olgahospital Stuttgart, Germany). All cancer cell lines were cultured in Dulbecco’s modified Eagle’s medium plus Ultraglutamine 1 (Lonza, Verviers, Belgium), with 10% fetal bovine serum (PAN Biotech GmbH, Aidenbach, Germany) and 1% Antibiotic-Antimycotic-Solution (Gibco, Paisley, UK). Primary human skeletal myoblasts (HSkM) were purchased from Gibco® (Cat. # A11440) and grown in Skeletal Muscle Cell Growth Medium including a Medium Supplement Pack (both from Promocell, Heidelberg, Germany) with 0.05 mg/ml fetal calf serum, 50 μg/ml fetuin (bovine), 10 ng/ml epidermal growth factor (recombinant human), 1 ng/ml basic fibroblast growth factor (recombinant human), 10 μg/ml insulin (recombinant human), and 0.4 μg/ml dexamethasone. For differentiation, HSkM cells were grown in Skeletal Muscle Differentiation Medium including 10 µg/ml insulin (recombinant human) from Promocell (Heidelberg, Germany) for at least 1 week. Cells were maintained at 37 °C and in a 5% CO_2_ atmosphere. Every second day the culture medium was changed, and confluent cancer cells were treated with 0.05% trypsin–0.02% EDTA (Lonza, Verviers, Belgium). Primary HSkM cells were treated with the Detach Kit (Promocell, Heidelberg, Germany) according to the manufacturer.

Cell lines RD, RH-30, A-240 were authetificated by the university of Tübingen, Children’s Hospital - Department of Pediatrics and HA-OH-1 and Ax-OH-1 by Cooperative soft tissue sarcoma-/ ‘CWS’- study group, Olgahospital Stuttgart, Germany. Morphology was checked by bright field microscopy. STR analysis was carried out to establish a DNA fingerprint by multiplex PCR. Mycoplasma detection was done by Hoechst 33258 staining.

### Transcript expression analysis

RNA isolation was done using the Aurum^TM^ Total RNA Mini Kit from Bio-Rad (USA) according to the protocol. cDNA synthesis was performed using RevertAid First Strand cDNA Synthesis Kit (#K1622) (Thermo Fisher Scientific Inc., Rockford, IL, USA) according to the product protocol. RT-PCR was performed as described previously [32] using the primer pairs listed in Table 1 and Dream Taq^TM^ Green PCR Master Mix (Thermo Fisher Scientific Inc., Vilnius, Lithuania) in the Eppendorf Mastercycler® ‘Mastercycler gradient’ (Eppendorf AG, Germany). Briefly, a 240 bp SGPL1 fragment in the C-terminal region of the SGPL1 coding sequence was amplified with primer pair fw: 5′-ATGCCTAGCACAGACCTTCT-3′ and rv: 5′-CTTCCTGGTGAGCTTAAAACA-3′.

**Table 1:** Overview and sequence of all used primer pairs for transcript amplification with RT-PCR

Name	Forward Primer	Reverse Primer
SGPL1 (Tv1)	5′-ATGCCTAGCACAGACCTTCT-3′	5′-CTTCCTGGTGAGCTTAAAACA-3′
SGPL1 (Tv all)	5′-ACTGCTCGCTTCCTCAAGTC-3′	5′-GTGACAGTGTCGGTGCTGTA-3′
GAPDH	5′-CAAGGTCATCCATGACAACTTTG-3′	5′-GTCCACCACCCTGTTGCTGTAG-3′
β-Actin	5′-GGGCATGGGTCAGAAGGATT-3′	5′-GAGGCGTACAGGGATAGCAC-3′
PAX3-FOXO1	5′-GCACTGTACACCAAAGCACG-3′	5′-CTGTGGATTGAGCATCCACC-3′
SPHK1	5′-TGGCGTCATGCATCTGTTCT-3′	5′-AGTAGTTTGGGTGCACCTGG-3′
SPHK2	5′-TCGTTCTGTGTCTGACCTGC-3′	5′-CATGAGCACAAAGTCCCCCT-3′
Ezrin	5′-TGCGGAGCTTGCAGAATACA-3′	5′-GGATGCCCTCACTAGACAGC-3′
CXCR4	5′-TCCATTCCTTTGCCTCTTTTGC-3′	5′-CCAGACGCCAACATAGACCA-3′
Myogenin	5′-GGTGCCCAGCGAATG-3′	5′-TGATGCTGTCCACGATCGA-3′

### Cloning and sequencing experiments

Sequencing of SGPL1 transcript variants was done by Seqlab Sequencing Laboratories (Göttingen, Germany). Forward and reverse sequencing was performed after amplification of SGPL1 coding sequence with the forward primer fw: 5′-ATGCCTAGCACAGACCTTCT-3′ and reverse primer: 5′-CTTCCTGGTGAGCTTAAAACA-3′ to determine alterations in the SGPL1 sequence. For determination whether the SGPL1 mutation in the coding sequence is homozygous or heterozygous, the purified SGPL1 PCR fragments generated by a thermostable *Taq* polymerase with proofreading function were cloned in the pGEM-T Vector *via* TA-cloning procedure using the pGEM®-T Easy Vector System II Kit (Promega Corp., Madison, USA) according to the manufacturer’s instructions. Briefly, the purified SGPL1 PCR fragments were A-tailed through dATP and *Taq* polymerase incubation for 15 min at 70 °C and further ligated into the linearized T-tailed pGEM®-T Easy Vector for 1 h at 24 °C. Afterwards the ligation product was transformed into JM109 competent *E.coli* cells. The competent cells were plated on LB/amp/IPTG/X-gal plates and the recombinant cells were identified by blue/white screening on indicator plates. Finally, ten SGPL1 recombinant clones were sequenced.

### Protein expression analysis

Western blot analysis was performed as already described [32, 40]. Briefly, for protein detection, primary antibodies anti-SGPL1 ((H-300) #sc-67368; Santa Cruz, USA), anti-SPHK1 ((M-209) #sc-48825; Santa Cruz, USA), anti-SPHK2 ((P-19) #sc-22704; Santa Cruz, USA), anti-PCNA ((PC10) #sc-56; Santa Cruz, USA), anti-β-Actin ((C4) #sc-47778; Santa Cruz, USA), anti-MTSS1 ((SS-3) #sc-101204; Santa Cruz, USA), anti-PARP1 ((B-10) #sc-74470; Santa Cruz, USA) and anti-Bcl-2 ((C-2) #sc-7382; Santa Cruz, USA) were incubated overnight at 4 °C followed by labeling with a horseradish peroxidase (HPR)-conjugated secondary antibody (mouse #7076; rabbit #7074P2; Cell Signaling, USA) for 1 h at room temperature. Finally, the protein signals were visualized with the Clarity™ Western ECL Chemiluminescent Substrate (Bio-Rad Laboratories Inc., USA). Stainfree-images and β-actin were used as loading control. Band intensity was analyzed densitometrically with the Molecular Imager ChemiDoc XRS and Image Lab 3.0.1 software (Bio-Rad, München, Germany). Protein detection was repeated at least three times with individually prepared cell lysates from independent passaged cells.

### Fluorescence microscopy

The fluorescence labeling procedure of HSkM, RH-30 and HA-OH1 cells was performed as described previously [32, 40]. Briefly, the cells were grown in Ibidi dishes/ slides (Ibidi GmbH, Martinsried, Germany), fixed in 4% paraformaldehyde (Santa Cruz, Dallas, USA), permeabilized with 0.1% Triton X-100 (Santa Cruz, Dallas, USA) and labeled with anti-SGPL1 primary antibody ((H-300) #sc-67368, Santa Cruz, USA) and Alexa Fluor 488 dye secondary antibody (Thermo Fisher Scientific Inc., USA). The co-localization experiments were performed by additional labeling with F-Actin antibody Phalloidin-Alexa 596 (Invitrogen, USA), focal adhesion kinase (FAK) primary antibody (#3285, Cell Signaling, USA) or with cell-permanent ER-Tracker™ Green dye (BODIPY® FL glibenclamide, Molecular Probes, USA). All samples were also counter-stained with Hoechst (PanReacAppliChem, Darmstadt, Germany). Images concerning on SGPL1 expression and localization were captured on a confocal laser-scanning microscope Leica DMi8 (Leica, Germany). Transfection efficiency (GFP signal of SGPL1; TYE-563 positive siRNA control) was controlled with a fluorescence microscope CKX53 (Olympus, Japan).

### *SGPL1*-plasmid/ -siRNA mediated overexpression and knockdown experiments

The SGPL1-cDNA-GFP-tagged clone including SGPL1 transcript variant 1 (#RG208705; NCBI Accession: NM_003901, NP_003892.2), 3 unique 27mer SGPL1-siRNAs (#SR305866; SGPL1 Human siRNA Oligo Duplex (Locus ID 8879)), Trilencer-27 Universal Scrambled Negative Control siRNA Duplex (#SR30004) and Trilencer-27 Fluorescent-labeled transfection control siRNA duplex (#SR30002) were all purchased from Origene (Rocville, USA). The plasmid map is shown in S. Fig. 4a. RH-30 and HA-OH1 cells (1 × 10^6^ cells) were transfected with 2.5 μg SGPL1 plasmid DNA or with 1 nM siRNA using the Lipofectamine™ 3000 Transfection Reagent (Invitrogen by Thermo Fisher Scientific, Carlsbad, USA) according the manufacture’s protocol. After 24 h transfection efficiency was controlled with fluorescence microscopy (microscope CKX53, Olympus, Japan) and GFP negative cells were eliminated by fluorescence-based cell sorting (MoFlo^TM^ Astrios, Beckman Coulter GmbH, Krefeld, Germany) (representative gating strategy is shown in S. Fig. 4b).

### Migration assays

Influence of 1 µM S1P or DHS (S1P #sc-201383 and DHS #sc-211174, Santa Cruz, USA) stimulation on RMA migration compared to the control (vehicle, MeOH) was first conducted with non-transfected RH-30 and HA-OH1 cells (Fig. 3c). Afterwards the migration capacity of stable SGPL1 and SGPL1 siRNA transfected RH-30 cells was analyzed under 1 µM S1P-stimulation (Fig. 5c). The Migration assay was performed with Ibidi culture inserts (μ-Dish 35 mm; Ibidi GmbH, Martinsried, Germany) according to the Ibidi protocol and gap closure was analyzed as described previously [32, 40, 49]. A pre-incubation in assay medium (DMEM, 10% charcoal stripped fetal bovine serum (PAN BiotechGmbH, Germany) for 48 h adaption in 6-well plates (Sarstedt, Germany) was done before every experiment and the medium was changed every day under treatment conditions. Images during gap closure were taken with the bright field microscope (CKX53, Olympus, Japan) and the gap area [µm^2^] was evaluated with the software CellSens Entry (Olympus, Germany).

### Cell viability and cytotoxicity

The cell viability and LDH release in course of S1P or DHS stimulation (extract concentration: 1 µM, 100 nM, 10 nM S1P and 10 µM, 1 µM, 100 nM, 10 nM DHS) of RH-30 and HA-OH1 cells compared to the control vehicle (MeOH) was quantified with the CellTiter 96®AQueous One Solution Cell Proliferation Assay Kit (MTS) (Promega Corp., Madison, USA) and Cytotoxicity Detection Kit (LDH) ‘Version 10’ (Roche Diagnostics GmbH, Mannheim, Germany) according to the manufacturer’´s instruction manuals as described previously [40, 49–51]. At least, eight replicates with corrected background absorbance were conducted. The cells were pre-incubated as well as treated in assay medium (DMEM, 10% charcoal stripped fetal bovine serum (PAN BiotechGmbH, Germany) for 48 h in the 96-well plates (Sarstedt, Germany). MTS and LDH assays were read using MRX Revelation 4.06 microplate reader (Dynex Technologies, USA).

### Adherent colony formation

Adherent colony formation during 1 µM S1P stimulation was performed according to the protocol of Franken et al. [52]. Briefly, stabile SGPL1 as well as SGPL1 siRNA transfected RH-30 cells were seeded at a density of 1 × 10^2^, −10^3^, and −10^4^ cells per well in 12-well plates and maintained for 10–14 days. Finally, cells were fixed and stained for 30 min with 6% glutaraldehyde (Santa Cruz, Dallas, USA) and 0.5% crystal violet (Sigma Aldrich, Saint Luis, USA). The images of colonies were taken with the bright field microscope (CKX53, Olympus, Japan). Colonies containing more than 25 cells were counted.

### SGPL1 surface expression analysis by flow cytometry

The fluorescence labeling procedure for SGPL1 plasma membrane association analysis by flow cytometry was conducted as described previously [32, 53]. In short, the polyclonal rabbit anti-SGPL1 antibody, recognizing a C-terminal epitope: amino acid 131–430 (cytoplasmic domain) ((H-300) #sc-67368, Santa Cruz, USA) was used in a 1:50 dilution. Secondary antibody (Alexa Fluor 488 dye; Thermo Fisher Scientific Inc., USA) was diluted 1:100.

### Statistical analysis

Western blotting, RT-PCR’s and Immunofluorescence experiments were replicated at least three times with individually passaged cells, and data sets were expressed as means ± standard deviations (SD). Statistically significant differences were compared using the unpaired Student’s *t*-test. *P* values: ***P < 0.001; **P < 0.01; *P < 0.05 were considered statistically as significant. All analyses were performed with the software Microsoft Excel 2017 and Graphpad Prism Version 5 (http://www.graphpad.com/scientific-software/prism/).

## Supplementary information

Supplemental Legends

Supplemental Material
