# Supplementary material for: SGPL1321 mutation: one main trigger for invasiveness of pediatric alveolar rhabdomyosarcoma
Source: Cancer Gene Ther. 2019 Aug 27;27(7):571–84. doi: 10.1038/s41417-019-0132-8 (PMC7445884; doi:10.1038/s41417-019-0132-8)
Supplement: Supplementary file 1 — Supplemental Legends [file 41417_2019_132_MOESM1_ESM.docx]

# **Supplemental Legends**

**S. Fig. 1** Stain-free image technique functioning as loading control. **a:** 10 μg protein per lane were applied in expression analysis experiments in Fig. 1b. **b:** 20 μg protein per lane were applied in the SGPL1 overexpression experiments in Fig. 5b. **c:** Representative gating strategy and antibody dilution series for the determination of SGPL1 association with the plasma membrane by flow cytometry of four RMS cell lines and HSkM cells compared to the positive MCF10A control. MCF10A control cells are known from Engel *et al.* (2018). **d:** Determination of SGPL1 association with the plasma membrane by flow cytometry of the RMS cell lines RD and Ax-OH-1 and the positive MCF10A control. All RMS cells in Figure 2b and S. Fig. 1d displayed no plasma membrane association of the SGPL1. In contrast, HSkM and positive control cells showed a positive signal for SGPL1 plasma membrane association. **e:** Immunofluorescence-based analysis of the different localization and distribution pattern of the mutated SGPL1 (red fluorescence) and native SGPL1 (restored SGPL1: green GFP signal) in RH-30 cells. Note, the SGPL1 variants are not co-localized.

**S. Fig. 2** Prescreening results of native SGPL1-restauration by stabile SGPL1 transfection in RH-30 and HA-OH1 cells. **a:** SGPL1 transcript level was significantly increased after SGPL1 transfection. The analysis was performed without elimination of non-transfected cells. **b:** SGPL1 protein content was significantly increased after SGPL1 transfection. The proliferation marker PCNA and metastasis marker MTSS1 were downregulated after restoration of the SGPL1 activity. The analysis was performed without elimination of non-transfected cells. **c:** Native SGPL1 overexpression significantly increased the SGPL1 transcript level and decreased SPHK1/2 isoenzyme as well as ezrin (metastasis marker) transcript level. The analysis was performed with positive SGPL1 transfected and GFP-sorted RH-30 cells. **d:** Native SGPL1 overexpression revealed another SGPL1 isoform (shorter; 40 kDa). The analysis was performed with positive SGPL1 transfected and GFP-sorted RH-30 cells.

**S. Fig. 3** Prescreening results. Representative images of the results from plasmid-based SGPL1-restaurated RH-30 cells. **a:** Determination of effective SGPL1 siRNA´s in RH-30 cells. SGPL1 siRNA 1 and 3 were further used in the experiments. **b:** RT-PCR of SGPL1, GAPDH and β-actin loading control as well as PAX3-FOXO1 RMA marker transcript expression in stable and transient SGPL1 overexpressors. Dark green SGPL1 marking represents GFP-sorted and light green marking GFP-unsorted SGPL1 transfected RH-30 cells. **c:** SGPL1 overexpression induced PARP-1 cleavage and reduced Bcl-2 overexpression and so revealed apoptosis induction. Whereas SGPL1 treatment with specific siRNA´s increased normal PARP-1 expression and Bcl-2 overexpression (anti-apoptotic effect). The analysis was performed with positive SGPL1 transfected and GFP-sorted RH-30 cells.

**S. Fig. 4 a:** Plasmid map of the SGPL1-ORF expression vector (#RG208705; Origene, Rocville, USA; https://www.origene.com/drawmapbysku?SKU=RG208705). **b:** Representative gating strategy for fluorescence-based cell sorting of positive SGPL1 transfected RH-30 cells. **c:** Representative control images of positive SGPL1-GFP transfected cells. The GFP signal was visualized by fluorescence microscopy.

**S. Fig. 5** Supporting pictures and bright field images of adherent colony formation assay under physiological S1P stimulation (1 µM) in Fig. 5d, after SGPL1-restauration and siRNA mediated SGPL1 knockdown in RH-30 cells.

**S. Fig. 6: a:** Summary of results from Kaplan-Meier scanner with the online R2 correlation analysis tool (https://hgserver1.amc.nl/cgi-bin/r2/main.cgi) displaying the overall survival probability in dependence of high SGPL1 expression for 13 different cancer entities. (negative correlation is marked with – and positive correlation with +). **b:** Correlation analysis of positive pax-foxo1 fusion chimere expression and SGPL1 overexpression in rhabdomyosarcoma cells. The analysis was performed the online R2 correlation analysis tool (https://hgserver1.amc.nl/cgi-bin/r2/main.cgi).

**S. Fig. 7 – 10:** Uncropped gels and blots.
