# Supplementary material for: SGPL1321 mutation: one main trigger for invasiveness of pediatric alveolar rhabdomyosarcoma
Source: Cancer Gene Ther. 2019 Aug 27;27(7):571–84. doi: 10.1038/s41417-019-0132-8 (PMC7445884; doi:10.1038/s41417-019-0132-8)

Supplemental Figure 1

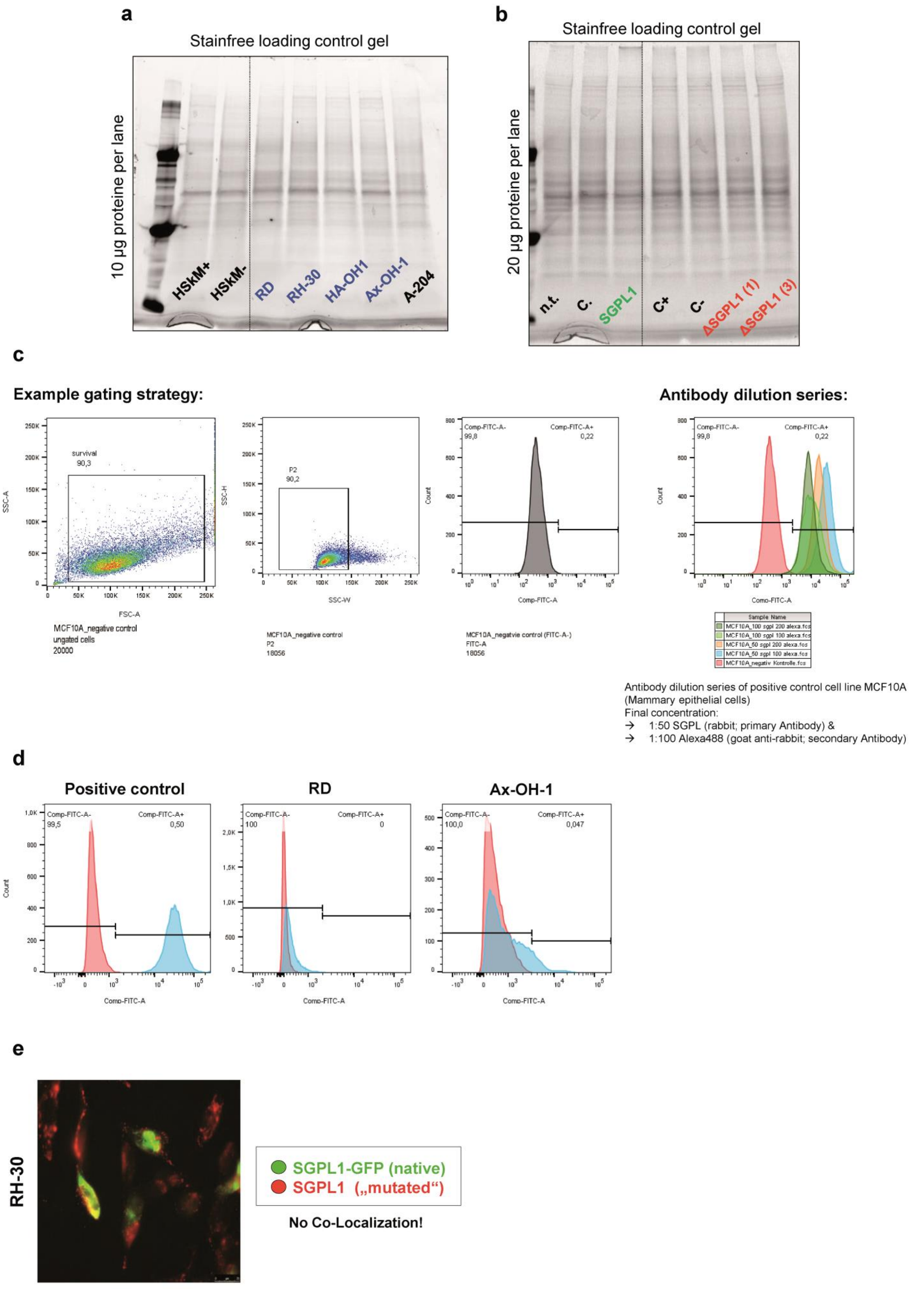

Supplemental Figure 2

Plasmid based SGPL1 transfection

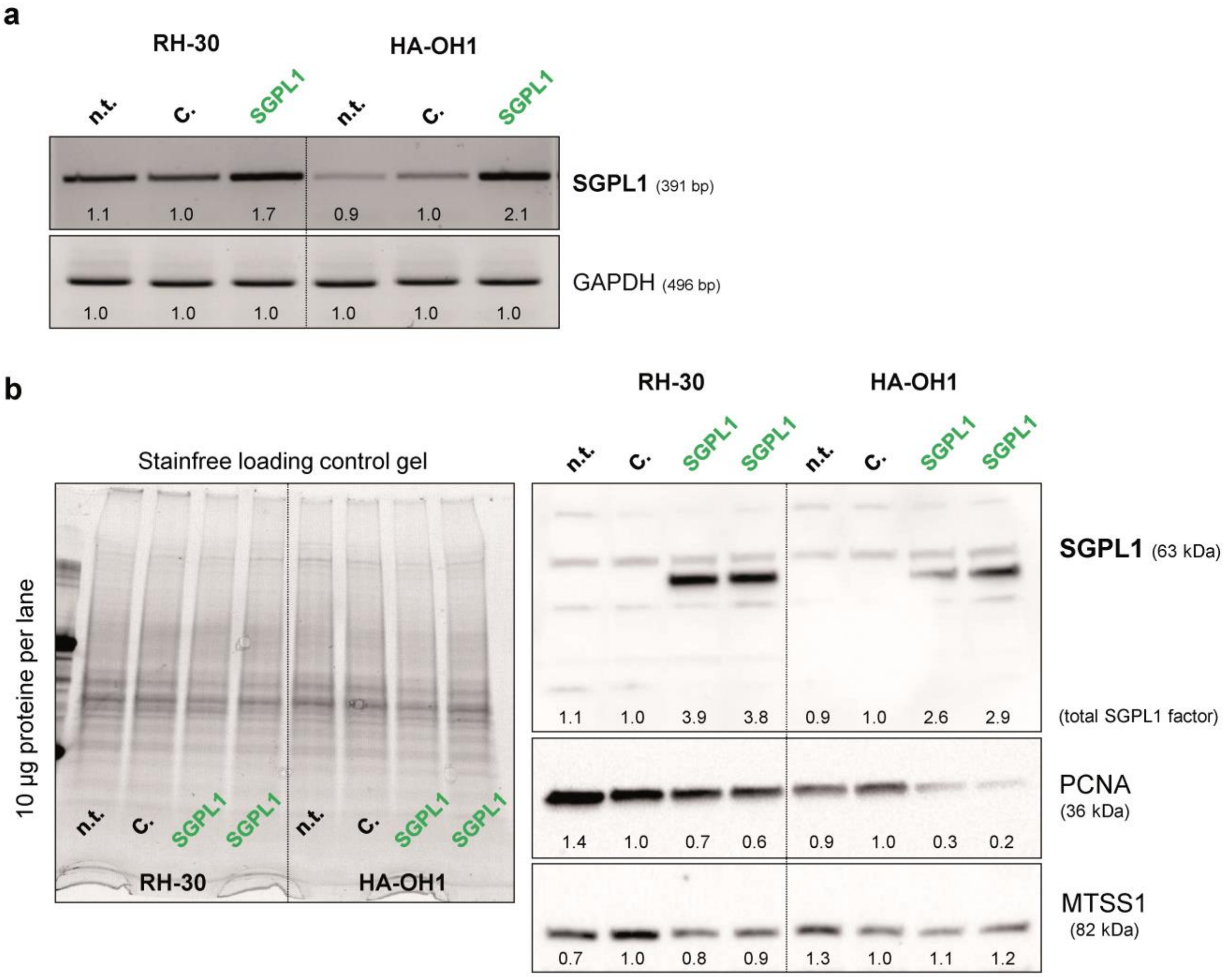

Figure a & b: Images with GFP-unsorted SGPL1 transfected alveolar RMS cell line (RH-30 & HA-OH1)

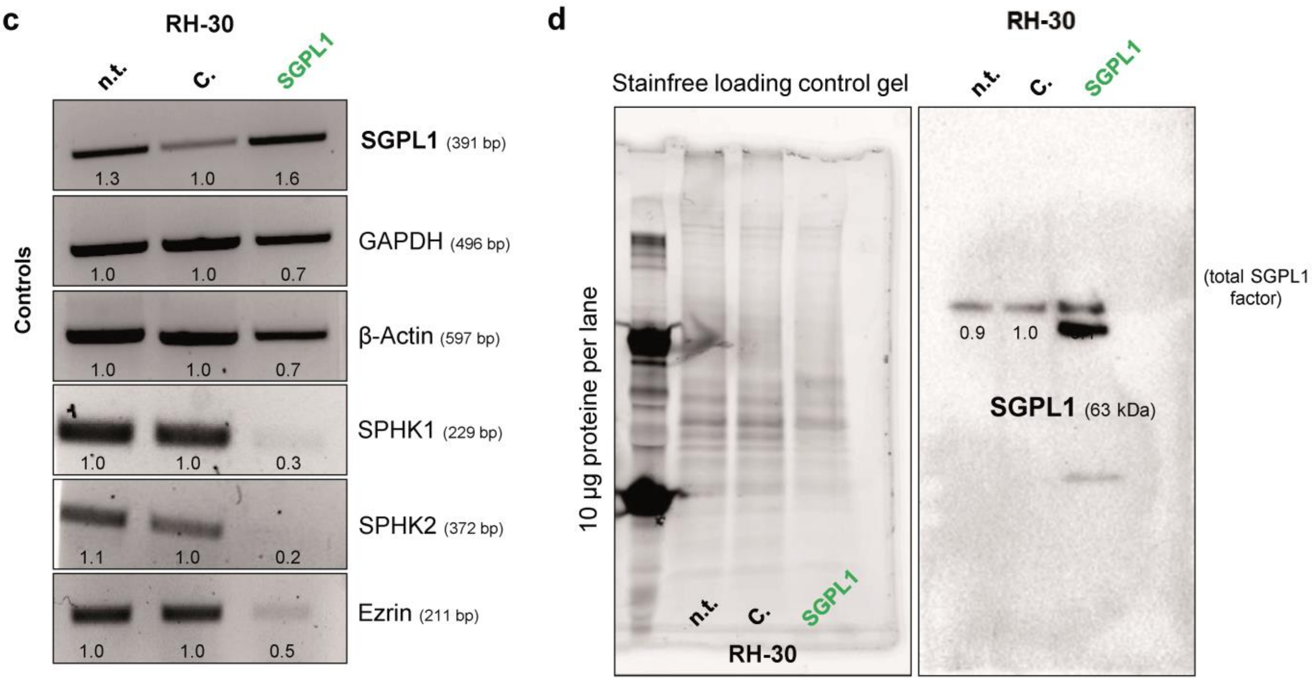

Supplemental Figure 3

SGPL1 siRNA screening

a

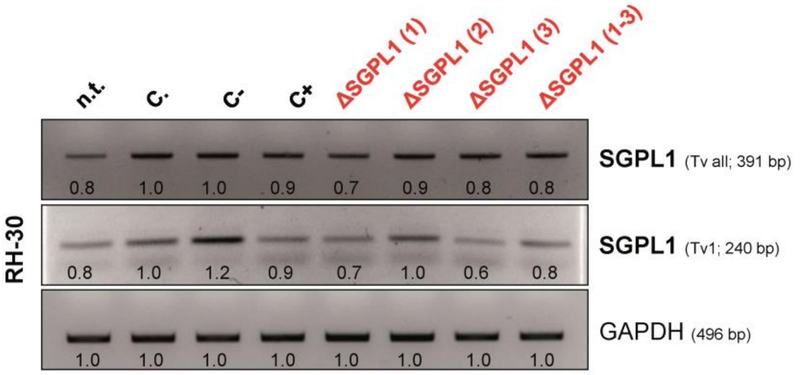

→ **ΔSGPL1 siRNA 1 & 3:** were chosen for further experiments

b

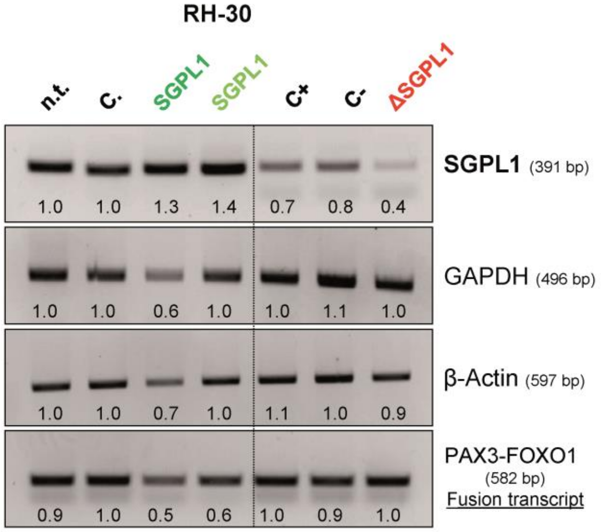

Figure b: Images with GFP-sorted (left) and -unsorted (right) SGPL1 transfected alveolar RMS cell line (RH-30)

c

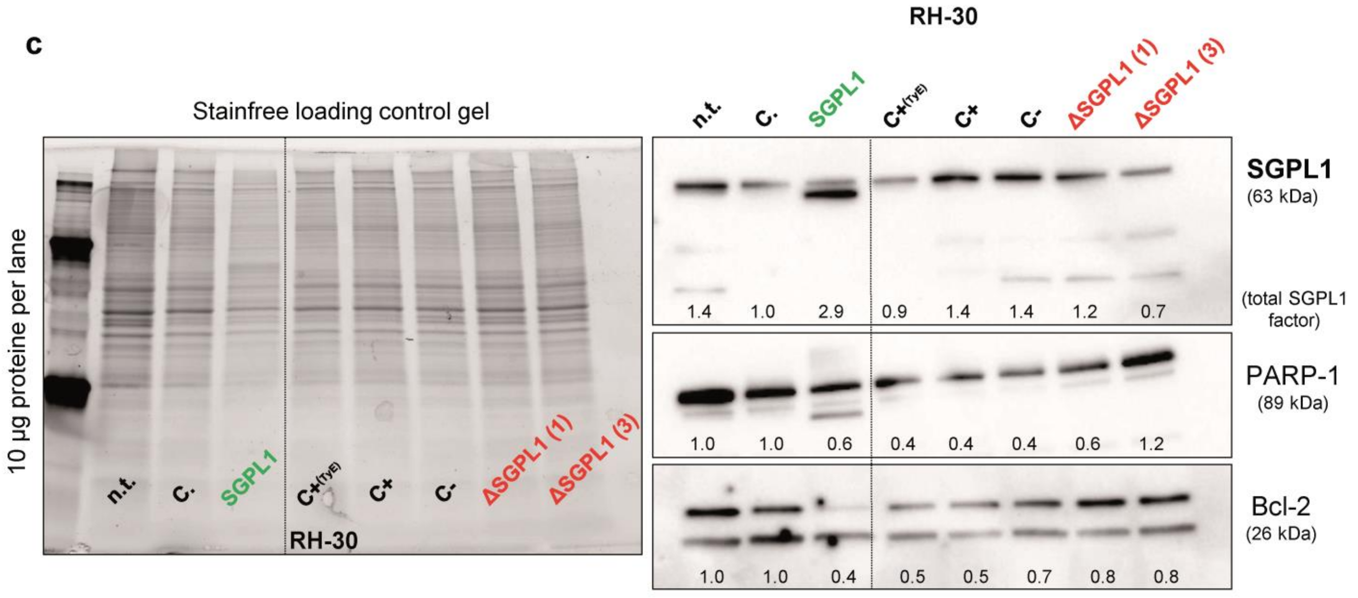

Figure c: Images with GFP-sorted SGPL1 transfected alveolar RMS cell line (RH-30)

Supplemental Figure 4

a

Circular map for RG208705:

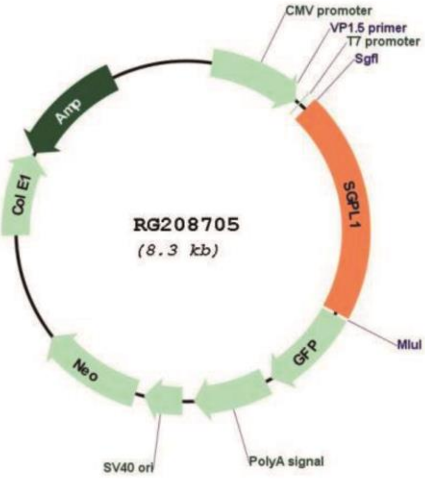

b

Example gating strategy:

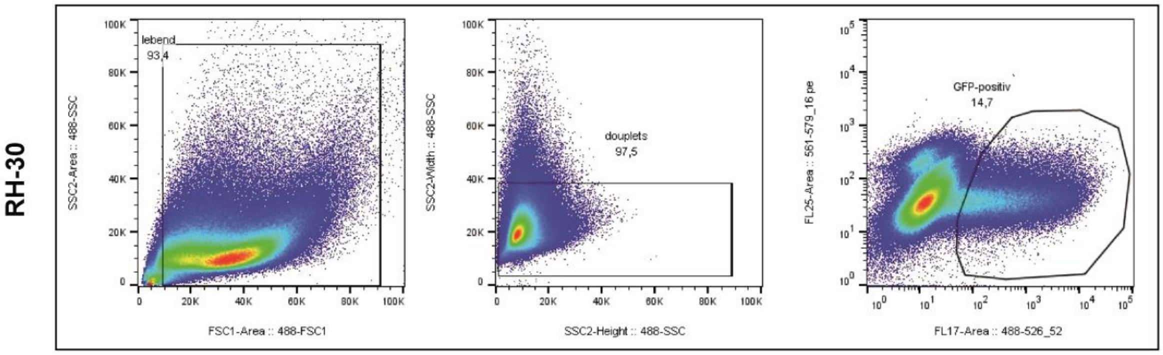

c

SGPL1-GFP

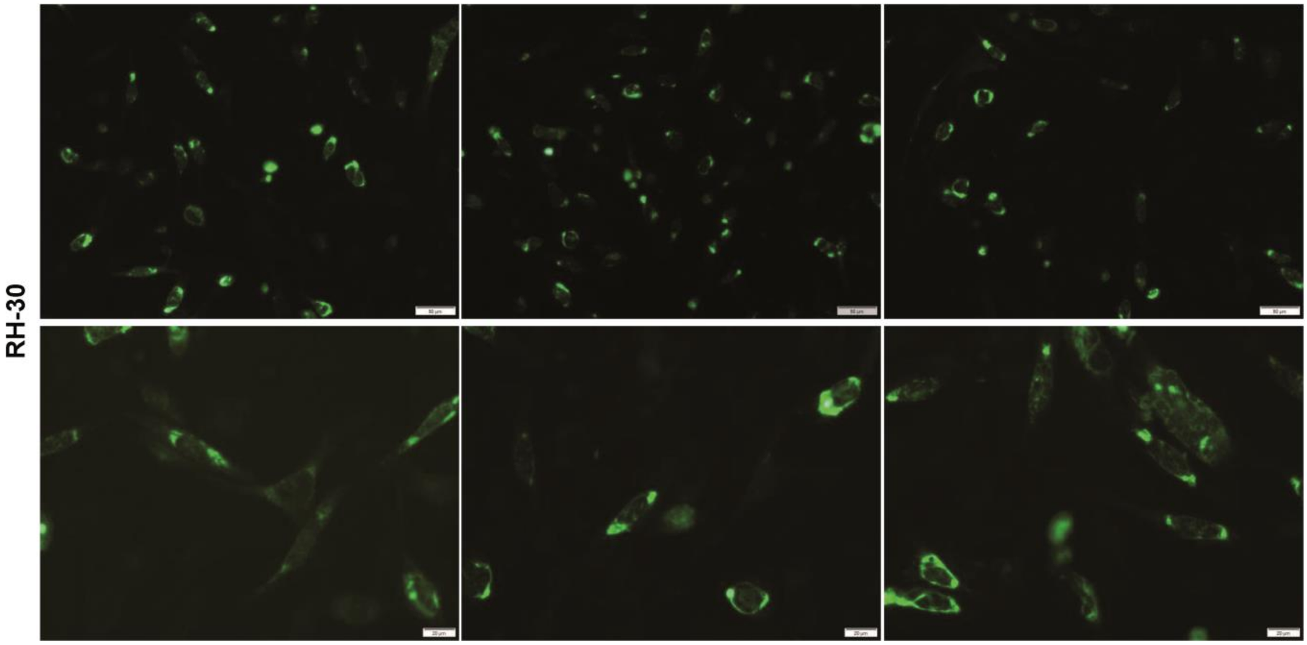

Supplemental Figure 5

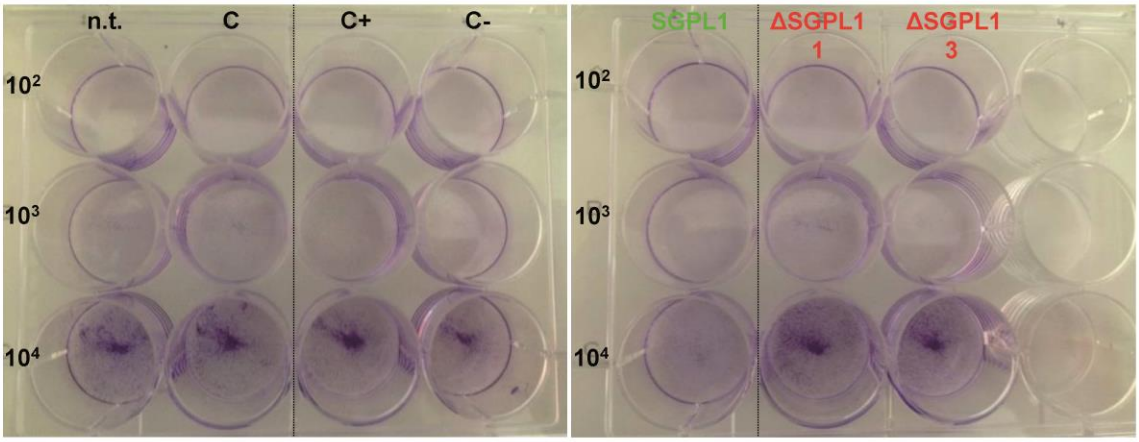

RH-30 cells were defined (10^2, 10^3, 10^4 cells/ well) seeded 48 h after the transfection.
Images were taken after 14 days. (See the graphicial evaluation in Figure 5b)

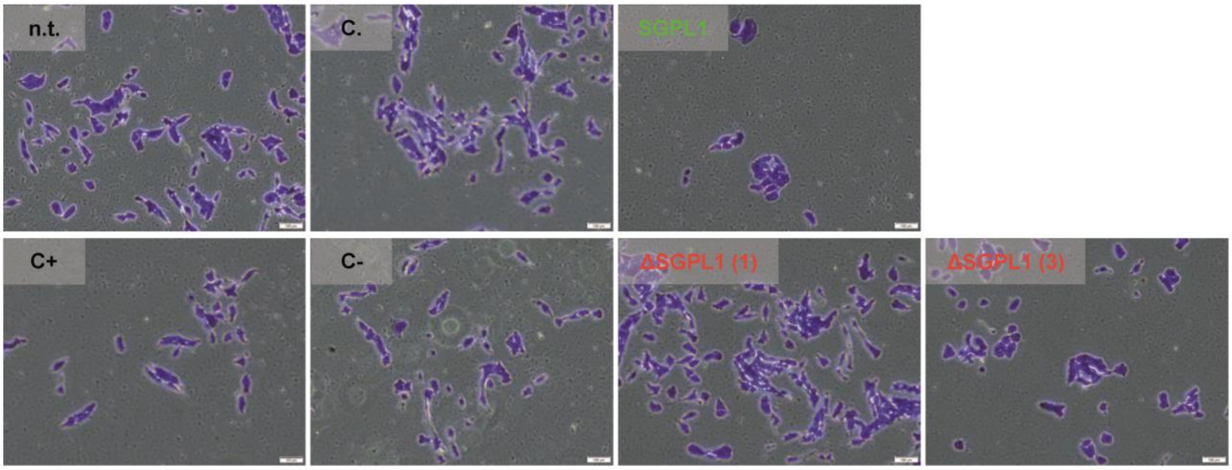

Supplemental Figure 6

a

Tumor Type	High level of SGPL1<br>- correlation with overall survival -
Neuroblastoma<br>(Versteeg-88; Capasso-27)	+
Wilms-Tumor<br>(OCG-125; OCG-148)	-
Ewing Sarcoma<br>(Savola-117; Dirksen-85)	+
Rhabdomyosarcoma<br>(Barr-58)	-
Osteosarcoma<br>(Kuijer-127)	+
Breast Cancer<br>(Bergh-159; Clynes-121)	+
Ovarian Cancer<br>(Pamula Pilat-101; Mechta-Grigoriou-107)	+
Lung Cancer<br>(Bild-114)	+
Esophagal Carcinoma<br>(TCGA-184)	-
B-Cell Lymphoma<br>(Xiao-420)	-/+
T-Cell Lymphoma<br>(Rosenwald-193)	-
Metastatic Melanoma<br>(Bhardwaj-44)	+
Glioblastoma<br>(Hegi-84)	+

- = negative/ + = positive correlation with overall survival

b

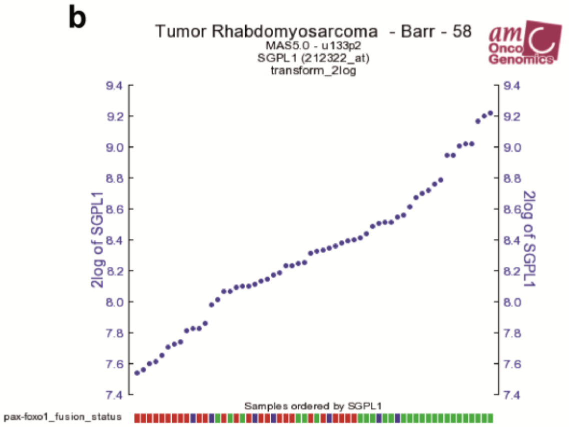

Positive pax-foxo1 fusion status correlate with SGPL1 overexpression in RMS.

Supplemental Figure 7

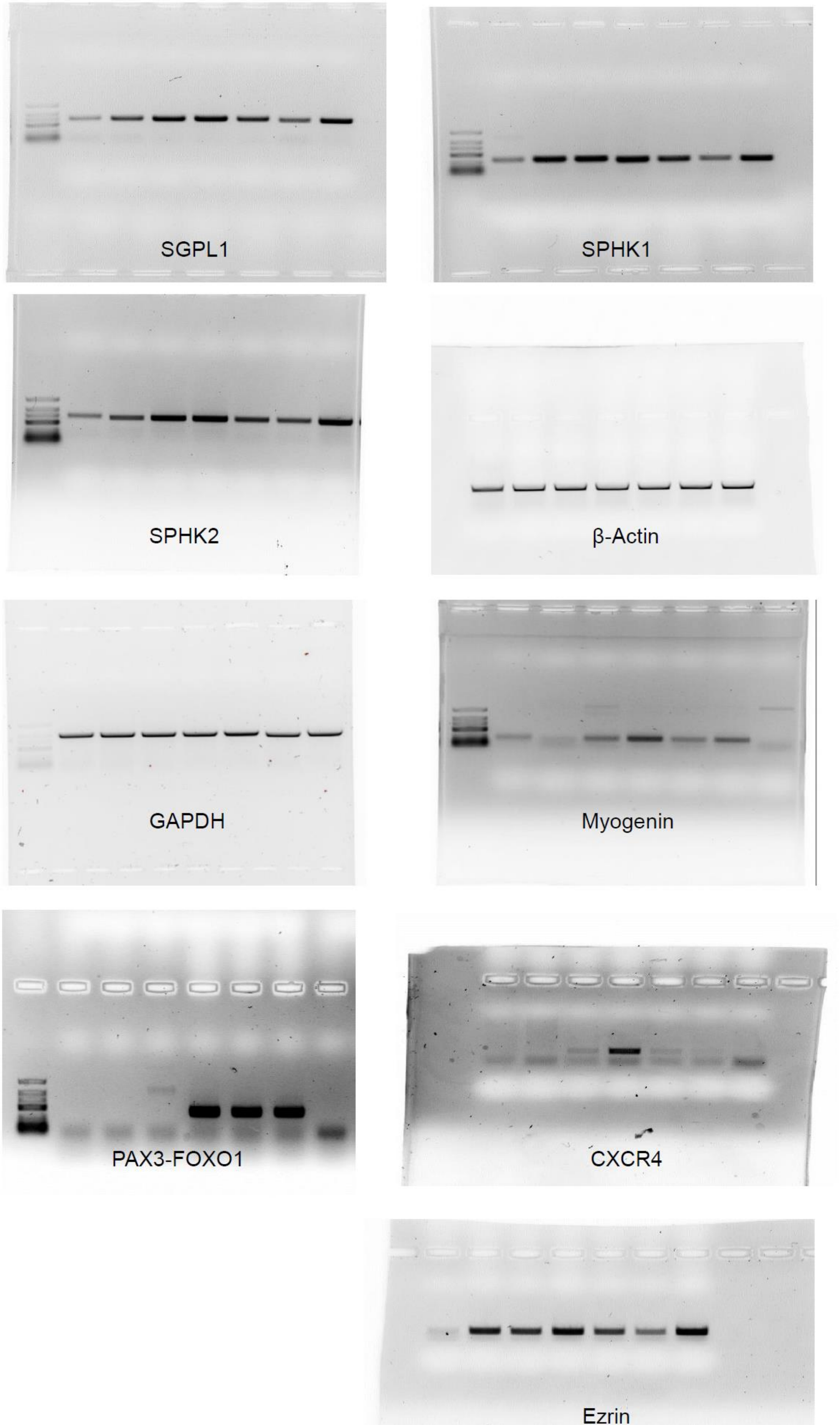

Supplemental Figure 8

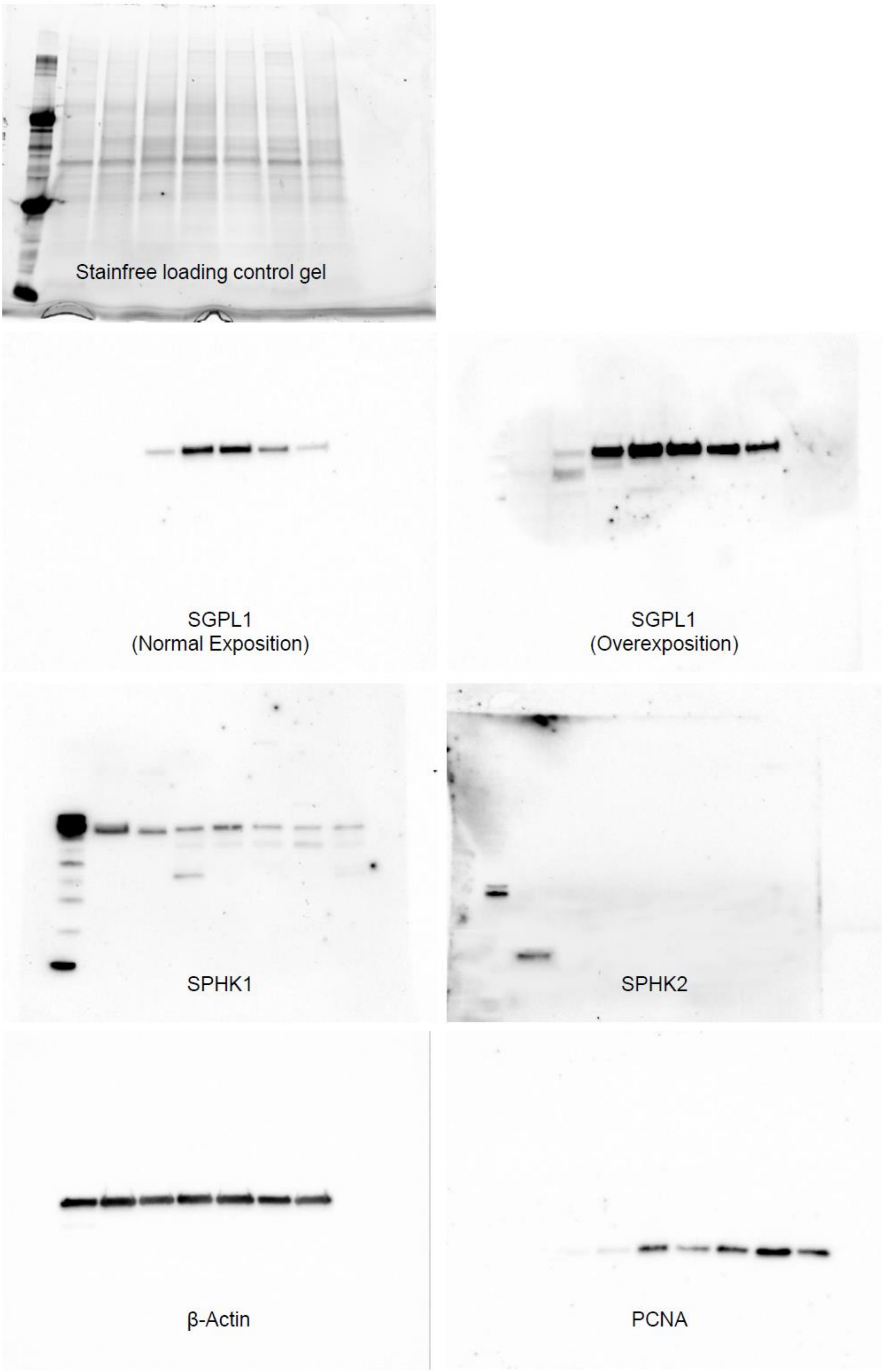

Supplemental Figure 9

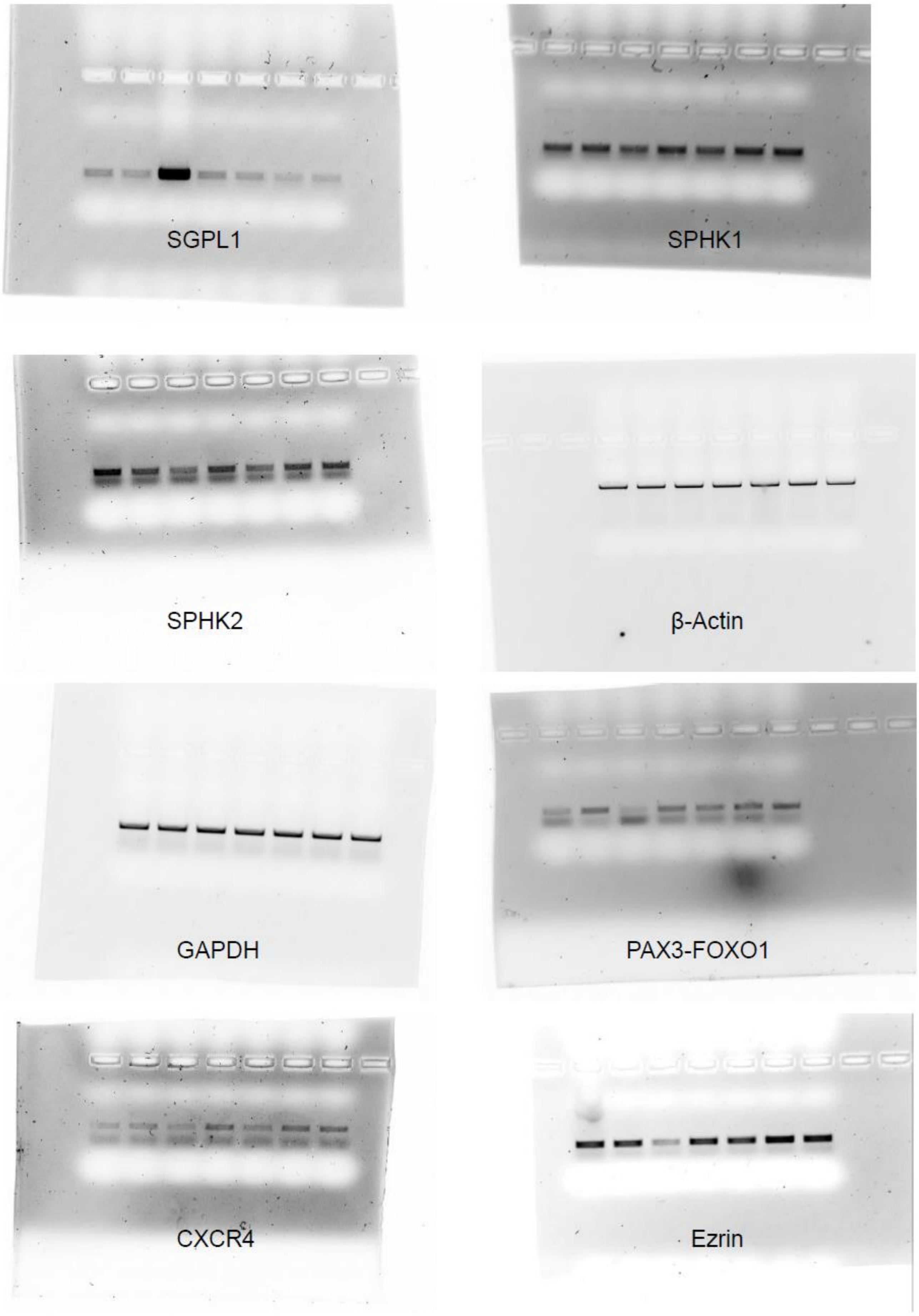

Supplemental Figure 10

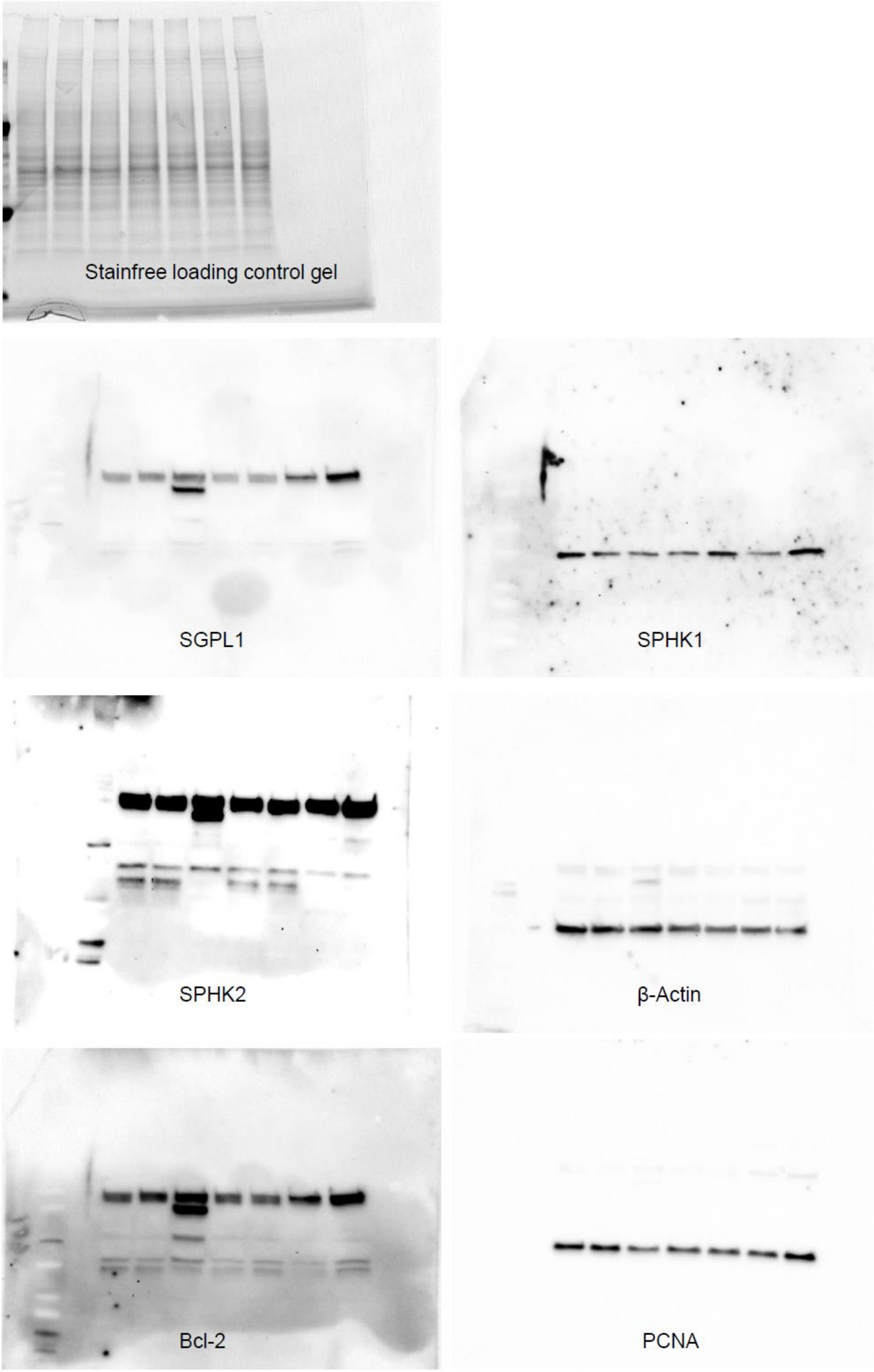

Supplement: Supplementary file 2 — Supplemental Material [file 41417_2019_132_MOESM2_ESM.pdf]
